# VCP/p97, a pleiotropic protein regulator of the DNA damage response and proteostasis, is a potential therapeutic target in *KRAS*-mutant pancreatic cancer

**DOI:** 10.18632/genesandcancer.231

**Published:** 2023-03-10

**Authors:** Ye S. Lee, Jennifer E. Klomp, Clint A. Stalnecker, Craig M. Goodwin, Yanzhe Gao, Gaith N. Droby, Cyrus Vaziri, Kirsten L. Bryant, Channing J. Der, Adrienne D. Cox

**Affiliations:** ^1^Department of Pharmacology, University of North Carolina at Chapel Hill, Chapel Hill, NC 27599, USA; ^2^Lineberger Comprehensive Cancer Center, University of North Carolina at Chapel Hill, Chapel Hill, NC 27599, USA; ^3^Department of Pathology and Laboratory Medicine, University of North Carolina at Chapel Hill, Chapel Hill, NC 27599, USA; ^4^Curriculum in Genetics and Molecular Biology, University of North Carolina at Chapel Hill, Chapel Hill, NC 27599, USA; ^5^Department of Biochemistry and Biophysics, University of North Carolina at Chapel Hill, Chapel Hill, NC 27599, USA; ^6^Department of Radiation Oncology, University of North Carolina at Chapel Hill, Chapel Hill, NC 27599, USA

**Keywords:** autophagy, DNA damage response, KRAS, pancreatic cancer, VCP

## Abstract

We and others have recently shown that proteins involved in the DNA damage response (DDR) are critical for *KRAS*-mutant pancreatic ductal adenocarcinoma (PDAC) cell growth *in vitro*. However, the CRISPR-Cas9 library that enabled us to identify these key proteins had limited representation of DDR-related genes. To further investigate the DDR in this context, we performed a comprehensive, DDR-focused CRISPR-Cas9 loss-of-function screen. This screen identified valosin-containing protein (*VCP*) as an essential gene in *KRAS*-mutant PDAC cell lines. We observed that genetic and pharmacologic inhibition of VCP limited cell growth and induced apoptotic death. Addressing the basis for VCP-dependent growth, we first evaluated the contribution of VCP to the DDR and found that loss of VCP resulted in accumulation of DNA double-strand breaks. We next addressed its role in proteostasis and found that loss of VCP caused accumulation of polyubiquitinated proteins. We also found that loss of VCP increased autophagy. Therefore, we reasoned that inhibiting both VCP and autophagy could be an effective combination. Accordingly, we found that VCP inhibition synergized with the autophagy inhibitor chloroquine. We conclude that concurrent targeting of autophagy can enhance the efficacy of VCP inhibitors in *KRAS*-mutant PDAC.

## INTRODUCTION

Pancreatic ductal adenocarcinoma (PDAC) has a dismal 5-year survival rate of 11% [1] and is projected to become the second leading cause of cancer-related deaths in the US by 2030 [2]. Decades of research and countless efforts to develop treatment strategies for PDAC have yielded limited therapeutic options [3, 4]. Conventional cytotoxic drugs remain standard-of-care for PDAC [3, 5] despite the well-characterized genetic landscape [6] nominating therapeutic targets. Activating mutations of the *KRAS* oncogene occur in over 95% of PDAC [3, 7] and the role of KRAS in driving and maintaining PDAC growth is well-established. Recent advances have focused on the development of direct inhibitors targeting one KRAS mutation (Gly-12-Cys; G12C) [8, 9], one of which has been FDA-approved for lung cancer where KRAS^G12C^ mutations are the most prevalent [10]. There is also evidence of promising clinical activity of such inhibitors in PDAC [11, 12]; however, KRAS^G12C^ comprises less than 2% of KRAS mutants in PDAC [3]. Therefore, indirect strategies remain the best option for therapeutically targeting the majority of *KRAS*-mutant PDAC.

One potential anti-KRAS strategy is targeting pancreatic cancer cell dependence on proteostasis, or protein homeostasis, which maintains balance between protein translation and degradation [13]. Cancer cells are highly dependent on protein degradation pathways (i.e., autophagy and the ubiquitin-proteasome system [UPS]) due to their increased cellular metabolism [14, 15]. Autophagy is a crucial process by which cells degrade intracellular components to meet their energy requirements and maintain homeostasis. We and others have recently determined that inhibition of KRAS in *KRAS*-mutant PDAC cell lines increased dependence on autophagy compared to *KRAS*-wildtype PDAC cell lines [16, 17]. The UPS is an important system whereby polyubiquitinated proteins are shuttled to and degraded by the proteasome to mediate protein quality control and maintain proteostasis [18]. *KRAS* mutations have been shown to increase proteasome capacity and activity to promote cancer cell survival [14]. The dependence on both autophagy and UPS by *KRAS*-mutant cancer cells makes proteostasis dysregulation a convincing therapeutic approach.

Another promising anti-KRAS strategy targets the DNA damage response (DDR) [5], which is responsible for detecting and repairing DNA damage. The DDR also plays a critical role in regulating cellular processes including cell cycle progression, metabolism, and apoptosis [19]. The DDR activates the DNA damage checkpoint and utilization of DNA repair pathways to resolve DNA lesions inflicted by both endogenous and exogenous sources [20]. Cancer cells are highly dependent on the DDR to effectively resolve DNA lesions that occur as a result of the high levels of replication stress and genomic instability induced by oncogene activation and uncontrolled growth [21, 22]. Furthermore, some cancers have defective DNA repair proteins, rendering high-fidelity DNA repair (i.e., homologous recombination [HR]) difficult [23–25]. The resulting DNA damage repair deficiency exacerbates genomic instability and forces cancer cells to become heavily reliant on more error-prone repair pathways (e.g., non-homologous end-joining [NHEJ]) for survival [23–25]. Both genomic instability and DNA damage repair deficiency have been observed in PDAC [6, 26], making DDR proteins compelling therapeutic candidates [20].

These findings have led to the development of inhibitors that target components of the DDR to take advantage of the genomic instability and DNA damage repair deficiencies in a variety of cancer types [20, 27]. Recent studies, including our recent screens for drivers of *KRAS*-mutant PDAC, have shown that targeting components of the DDR (i.e., PARP, CHK1, WEE1, ATR, ATM) can effectively suppress PDAC growth [28–31]. In this study, we sought to identify additional DDR regulators as therapeutic targets for PDAC. To establish additional genes that are critical for PDAC growth, we designed and applied a DDR-focused library of sgRNAs to perform a CRISPR-Cas9 loss-of-function screen in *KRAS*-mutant PDAC cells and identified *VCP* as one such gene. *VCP* encodes VCP/p97, or valosin-containing protein, an ATPase that functions as a molecular chaperone, extracting ubiquitinated, misfolded client proteins from the endoplasmic reticulum (ER) for proteasomal degradation [32, 33]. VCP is a complex protein associated with numerous cellular activities including proteostasis (i.e., ubiquitin-proteasome system [UPS] and autophagy) and DNA damage repair [32, 34]. Elevated VCP expression has been observed in multiple cancer types, including PDAC, and correlates with worse patient survival [35–37].

While VCP has been heavily studied in the context of other cancer types, the mechanisms of VCP that modulate *KRAS*-mutant PDAC growth remain less well understood [37, 38]. We sought to determine the biological processes of VCP that contribute to the anti-proliferative and pro-apoptotic effects observed upon genetic and pharmacologic loss of VCP in *KRAS*-mutant PDAC. We showed that VCP is required for the degradation of polyubiquitinated proteins and that loss of VCP led to an increase in ER stress. We determined that VCP loss induced DNA damage, confirming the role that VCP plays in DNA damage repair. Interestingly, we found that VCP loss upregulated autophagic flux. Subsequently, we showed that dual pharmacological inhibition of VCP (CB-5083) and autophagy (chloroquine, CQ) enhanced growth suppression and apoptosis compared to either drug alone. We conclude that VCP is an important dependency of *KRAS*-mutant PDAC growth and that targeting VCP may be a useful therapeutic approach for KRAS-driven PDAC.

## RESULTS

### VCP is an essential gene for KRAS-mutant PDAC

**Figure 1:**
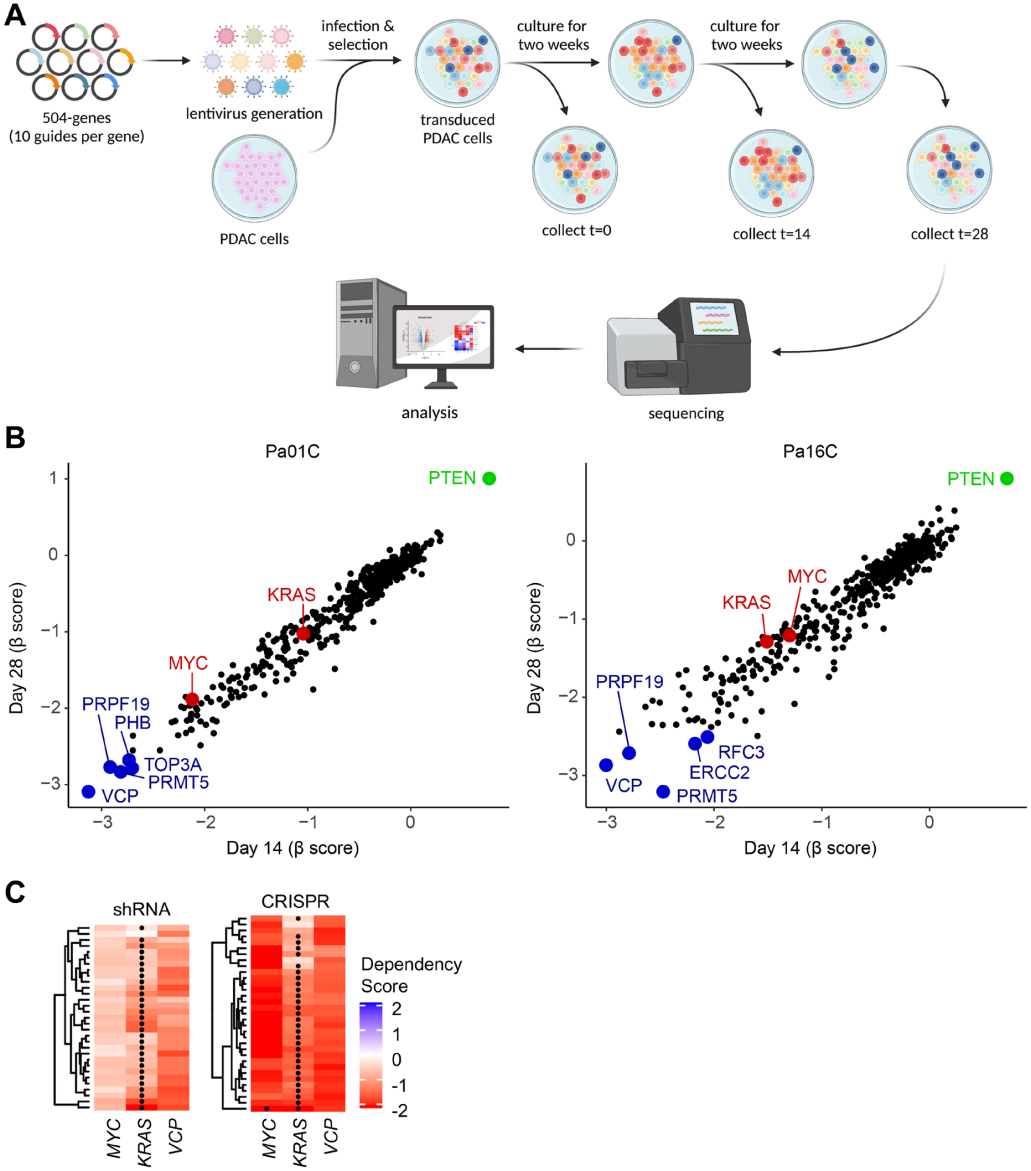
*VCP* is an essential gene for KRAS-mutant PDAC. (**A**) Schematic overview of the DNA damage response (DDR) loss-of-function CRISPR screen. (**B**) A loss-of-function CRISPR-Cas9 screen using a DNA damage response (DDR) library identified genes that inhibit cell proliferation in KRAS-mutant PDAC cell lines, Pa01C and Pa16C. MAGeCK MLE (maximum-likelihood estimation) analysis was used to obtain beta (β) scores, which measure the degree of selection upon gene loss. Negative β scores indicate that gene loss may inhibit cell proliferation or cause cell death. β scores for samples at 14 and 28 days are plotted. (**C**) Dependency of indicated genes from The Cancer Dependency Map (DepMap) for pancreatic cell lines using CRISPR or shRNA-mediated gene knockout. Each row of the heatmap corresponds to an individual cell line. Mutations of the indicated gene in a cell line are marked with a black dot. More negative scores indicate higher dependency on a gene for survival.

We recently determined that *KRAS*-mutant PDAC cell lines are heavily dependent on DNA damage response (DDR) pathways for cell growth [28]. In that study, we used a CRISPR-Cas9 loss-of-function library targeting 2,240 genes encoding major cellular and oncogenic signaling pathways, with only a limited representation of DDR genes [28, 39]. To further investigate the DDR in that context, we have now performed a loss-of-function CRISPR-Cas9 screen in the same *KRAS*-mutant PDAC cell lines, using a focused DDR library (Figure 1A) consisting of 504 DNA damage repair genes with 10 guides per gene. As expected, we observed that loss of the oncogenes *KRAS* and *MYC* impaired cell growth (Figure 1B). Also as expected, we observed that loss of the tumor suppressor *PTEN* enhanced cell growth (Figure 1B). In addition, we identified valosin-containing protein (*VCP*) as a gene essential for the viability of the *KRAS*-mutant PDAC cell lines (Figure 1B). Furthermore, we compared our CRISPR screen data to the Cancer Dependency Map (DepMap) CRISPR and shRNA screens from the Broad Institute [40]. All three data sets showed that PDAC cells have a high dependency on *KRAS*, *MYC* and *VCP* for growth (Figure 1C).

### VCP is required for PDAC growth

**Figure 2:**
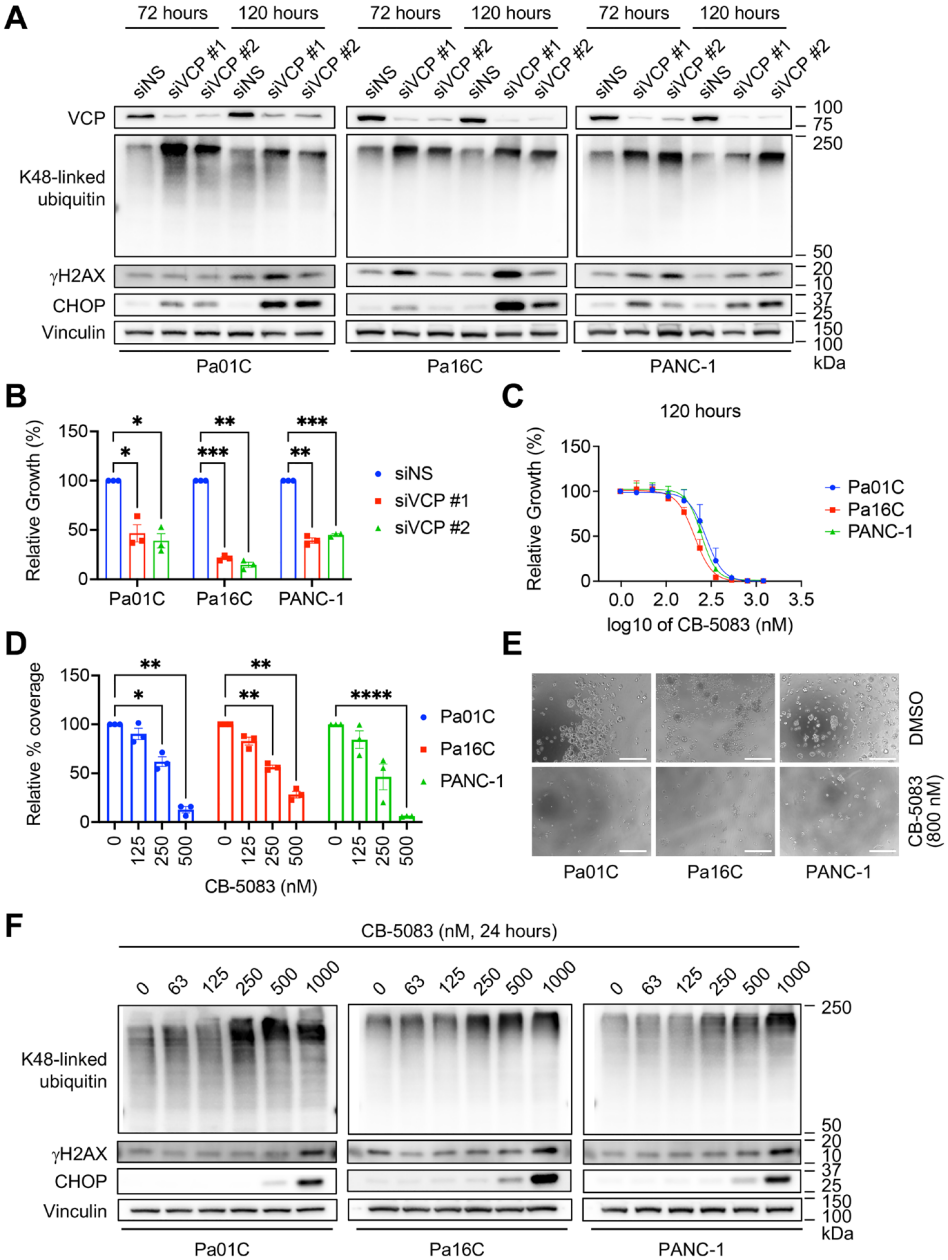
VCP is required for PDAC growth and polyubiquitinated protein degradation. (**A**) To determine the consequences of loss of VCP expression for the degradation of polyubiquitinated proteins, cells were reverse transfected for 72 and 120 hours with siNS, siVCP #1, or siVCP #2. Protein lysates were collected and immunoblot analyses were performed to determine the levels of K48-linked ubiquitin, γH2AX and CHOP. Vinculin served as a loading control. Figure is representative of three biological replicates. (**B**) To determine the importance of VCP to anchorage-dependent proliferation, cells were transiently transfected for 120 hours with siNS, siVCP #1, or siVCP #2. Proliferation was assessed at 120 hours by live cell counting. Mean cell counts were normalized to siNS control. Data shown are the mean ± SEM of three independent experiments. Two-way ANOVA followed by Dunnett’s multiple comparison test was used to determine statistical significance between the different groups. ^*^p < 0.05, ^**^p < 0.005, ^***^p ≤ 0.0005. Degree of knockdown is shown in Supplementary Figure 1B. (**C**) Cells were treated with a range of VCP inhibitor (CB-5083) concentrations (47–1200 nM) and proliferation was assessed at 120 hours by live cell counting. Data shown are the mean ± SEM of three independent experiments. (**D**) Anchorage-dependent colony-forming capacity was evaluated by staining with crystal violet after 10–14 days of treatment with vehicle control (0 nM CB-5083) or CB-5083 (125, 250, or 500 nM). Relative percent coverage was normalized to DMSO. Data shown are the mean ± SEM of three independent experiments. Two-way ANOVA followed by Dunnett’s multiple comparison test was used to determine statistical significance between the different groups. ^*^p < 0.05, ^**^p < 0.005, ^***^p < 0.0001. (**E**) Anchorage-independent growth was determined by 3D colony formation in soft agar. Cells were seeded into agar, treated with a range of concentrations of CB-5083 for 14 days and imaged on an EVOS microscope. Images are representative of three biological replicates, each of which included three technical replicates. Images of intermediate inhibitor concentrations are depicted in Supplementary Figure 1K. Scale bar, 300 μm. (**F**) Cells were treated with VCP inhibitor CB-5083 for 24 hours, protein lysates were collected, and immunoblot analyses were performed to determine the levels of indicated proteins as in panel A. Figure is representative of three biological replicates.

To explore the requirement for VCP in cell growth, we investigated the consequences of acute siRNA-mediated *VCP* suppression in *KRAS*-mutant PDAC cell lines. We monitored *VCP* suppression by immunoblotting for VCP itself (Figure 2A). PDAC cell proliferation was reduced at timepoints over 120 hours (Figure 2B, Supplementary Figure 1A) upon *VCP* suppression (Supplementary Figure 1B). Additionally, clonogenic growth of *KRAS*-mutant PDAC cell lines was reduced (Supplementary Figure 1C, 1D) upon *VCP* suppression (Supplementary Figure 1E). As a complementary method of evaluating the requirement for VCP and to address potential off-target activities of siRNA-mediated VCP suppression, we obtained the pharmacological VCP inhibitor (VCPi) CB-5083 [41], the best commercially available translational VCPi for preclinical studies. VCP inhibition at 72 hours (Supplementary Figure 1F) and 120 hours (Figure 2C) was sufficient to reduce anchorage-dependent proliferation in a dose-dependent manner, with a GI_50_ of ~250 nM across timepoints and cell lines (Supplementary Figure 1G). Similar growth inhibitory activities of VCPi were seen in both anchorage-dependent clonogenic assays (Figure 2D, Supplementary Figure 1H) and anchorage-independent 3D colony formation in soft agar (Figure 2E, Supplementary Figure 1I–1K). These results support our conclusion that there is a high dependency on VCP for *KRAS*-mutant PDAC growth.

### VCP is required for degradation of polyubiquitinated proteins

Given the importance of VCP in proteasomal protein degradation [32, 33], we investigated the consequences on protein degradation of acute siRNA-mediated *VCP* suppression in *KRAS*-mutant PDAC cell lines. We confirmed loss of VCP expression, and therefore loss of function, by immunoblotting for VCP itself and observed the accumulation of polyubiquitinated proteins (Figure 2A). Additionally, we noted increased levels of CHOP upon *VCP* suppression (Figure 2A), indicating activation of the unfolded protein response (UPR), which is triggered by high levels of ER stress [34]. To supplement our findings, we utilized the VCPi CB-5083. Concordant with the effects of genetic suppression, we observed dose-dependent accumulation of K48-linked ubiquitin and CHOP in PDAC cell lines treated with VCPi for 24 hours (Figure 2F). We conclude that VCP is required for the degradation of polyubiquitinated proteins to maintain proteostasis in *KRAS*-mutant PDAC.

### Loss of VCP induces programmed cell death in PDAC

**Figure 3:**
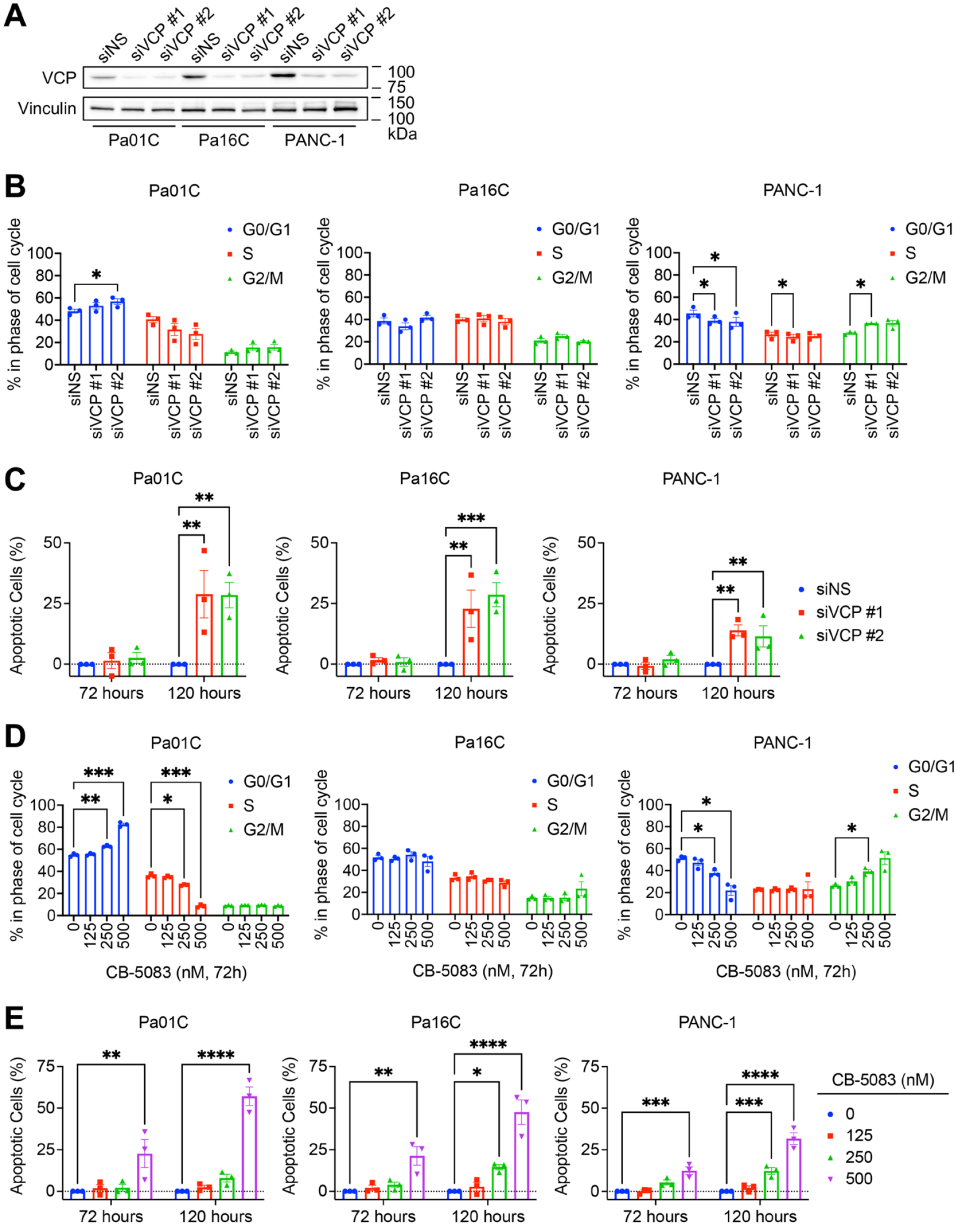
Loss of VCP induces PDAC cell death. (**A**) Cells were reverse transfected with non-specific (siNS) control or two different siRNAs targeting *VCP* (siVCP #1 or siVCP #2) for 72 hours. Immunoblot analyses were performed to determine knockdown efficiency. Representative of three biological replicates that correspond to panels B and C. (**B**) Percentage of cells in the specified phases of the cell cycle was determined by propidium iodide staining and flow cytometry following 72 hours of reverse transfection using siNS, siVCP #1, or siVCP #2. Data shown are the mean ± SEM of three independent experiments. Two-way ANOVA followed by Dunnett’s multiple comparison test was used to determine statistical significance. ^*^p < 0.05. (**C**) Cells were transiently transfected with siNS, siVCP #1, or siVCP #2 for 72 or 120 hours. Percentage of cells undergoing apoptosis was determined by FACS analysis of cells labeled with Annexin-V and propidium iodide. All treated populations were normalized to their respective siNS control. Data shown are the mean ± SEM of three independent experiments. Two-way ANOVA followed by Dunnett’s multiple comparison test was used to determine statistical significance. ^**^p < 0.0001 and ^***^p < 0.0005. (**D**) Percentage of cells in the specified phases of the cell cycle was determined and analyzed as in panel B, following 72 hours of treatment using CB-5083 (0, 125, 250, 500 nM). ^*^p < 0.05, ^**^p < 0.01 and ^***^p < 0.005. (**E**) Cells were treated with VCPi CB-5083 (125, 250, or 500 nM) for 72 or 120 hours. Percentage of cells undergoing apoptosis was determined and analyzed as in panel C, except that each respective control was 0 nM CB-5083, i.e., DMSO vehicle only. ^*^p < 0.05, ^**^p < 0.005, ^***^p < 0.001, and ^****^p < 0.0001.

We next sought to determine whether *KRAS*-mutant PDAC growth inhibition upon *VCP* loss was mediated through the induction of apoptosis and/or perturbation of cell cycle progression. We first determined that genetic suppression of *VCP* (siVCP) (Figure 3A) had variable effects on the cell cycle (Figure 3B). We observed cell cycle perturbations that were cell line dependent: G0/G1 arrest in Pa01C cells, no consistent effect in Pa16C cells, and G2/M arrest in PANC-1 cells. We then monitored the impact of *VCP* suppression on apoptosis at 72 and 120 hours. Correlating with our proliferation data (Supplementary Figure 1A), *VCP* suppression induced significant levels of apoptosis at 120 hours (Figure 3C, Supplementary Figure 2A).

We then evaluated cell cycle and apoptosis effects following pharmacological inhibition of VCP (CB-5083, 125–500 nM) in PDAC cell lines. Upon VCPi treatment for 72 hours, we observed the same modest cell line-dependent effects on the cell cycle (Figure 3D) as we had observed upon *VCP* suppression (Figure 3B). Apoptosis was induced at concentrations above the GI_50_ (Figure 3E, Supplementary Figure 2B). This effect was further enhanced at 120 hours of VCPi treatment (Figure 3E, Supplementary Figure 2B), also consistent with our *VCP* suppression data (Figure 3C). We conclude that PDAC growth suppression upon loss of VCP is mediated mainly by induction of apoptosis.

### VCP helps mediate DNA damage repair in PDAC

**Figure 4:**
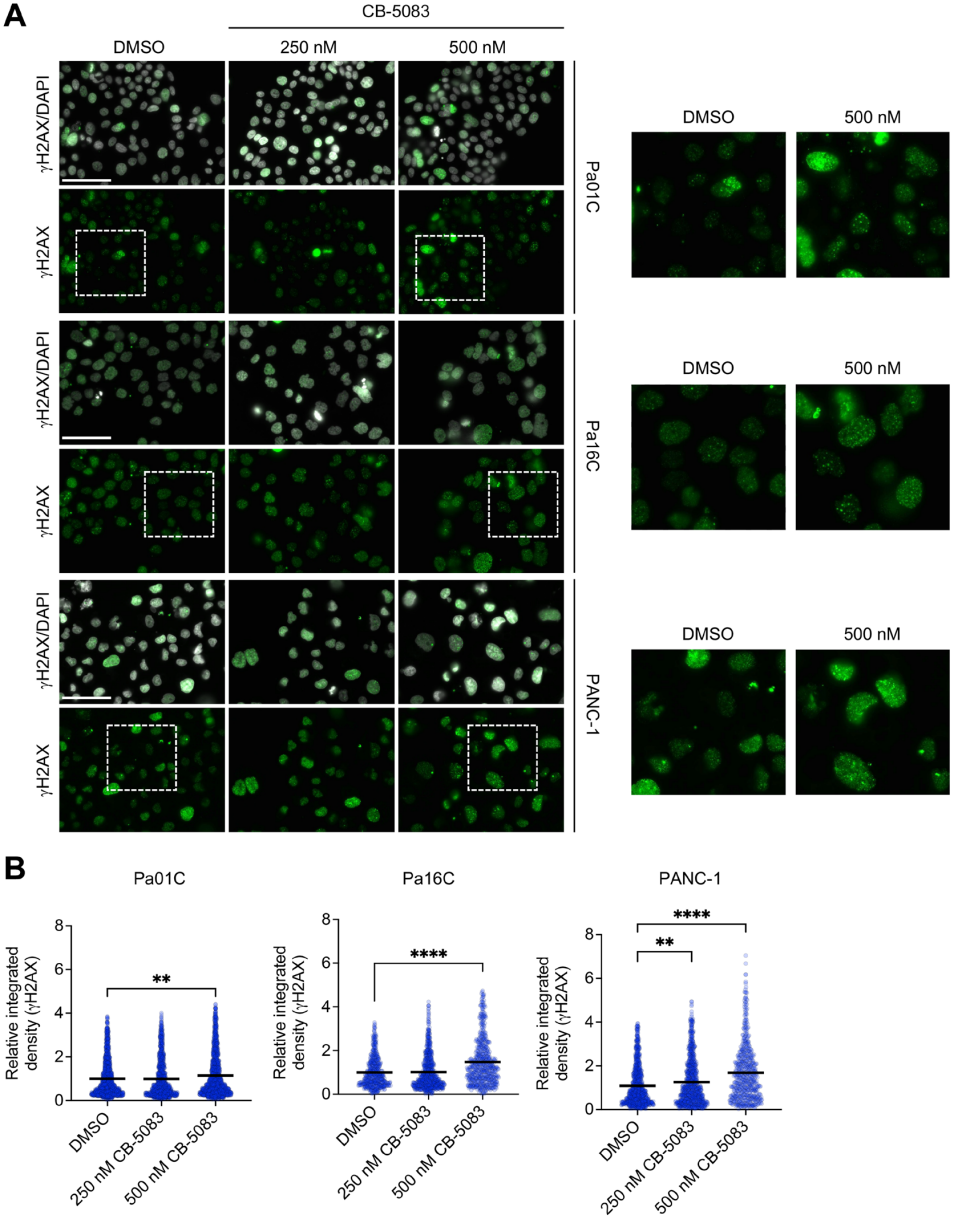
VCP, a regulator of DDR, helps mediate DNA damage repair in PDAC. (**A**) Representative images of immunofluorescence (IF) to monitor γH2AX expression (green) and nuclei (white) in PDAC cells after 24 hours of treatment with DMSO or CB-5083 at the indicated concentrations (nM). Scale bar, 75 μm. (**B**) Quantitation of relative integrated intensity of γH2AX per nucleus of cells shown in panel A. Each dot represents a nucleus; each bar indicates the mean of that treatment group. Statistical significance was calculated using one-way ANOVA and Kruskal-Wallis test relative to DMSO. ^**^p < 0.01 and ^****^p < 0.0001. The total number of nuclei analyzed for each cell line and condition (DMSO, 250 nM CB-5083, 500 nM CB-5083) were: Pa01C (1460, 1143, 1495), Pa16C (545, 746, 592), and PANC-1 (962, 902, 588), respectively.

Mutationally activated KRAS induces genotoxic stress, and the survival of cells expressing mutant KRAS depends on mechanisms that can mitigate the resulting DNA damage [42]. Because VCP regulates DNA double-strand break (DSB) repair, we investigated the consequences of VCP inhibition on DSB repair in a panel of *KRAS*-mutant PDAC cell lines. By immunoblotting for γH2AX, a marker of DSBs, we observed accumulation of DSBs upon genetic suppression or pharmacological inhibition of VCP (Figure 2A, 2F). As a complementary approach, we utilized immunofluorescence to determine the intensity of the γH2AX signal in the presence of the VCPi CB-5083. We found that the γH2AX signal intensity increased across all PDAC cell lines in a dose-dependent manner (Figure 4A, 4B, Supplementary Figure 3). Our data indicate that both genetic suppression and pharmacologic inhibition of VCP impedes the ability of PDAC cells to resolve DNA damage.

### VCP loss elevates autophagic flux

**Figure 5:**
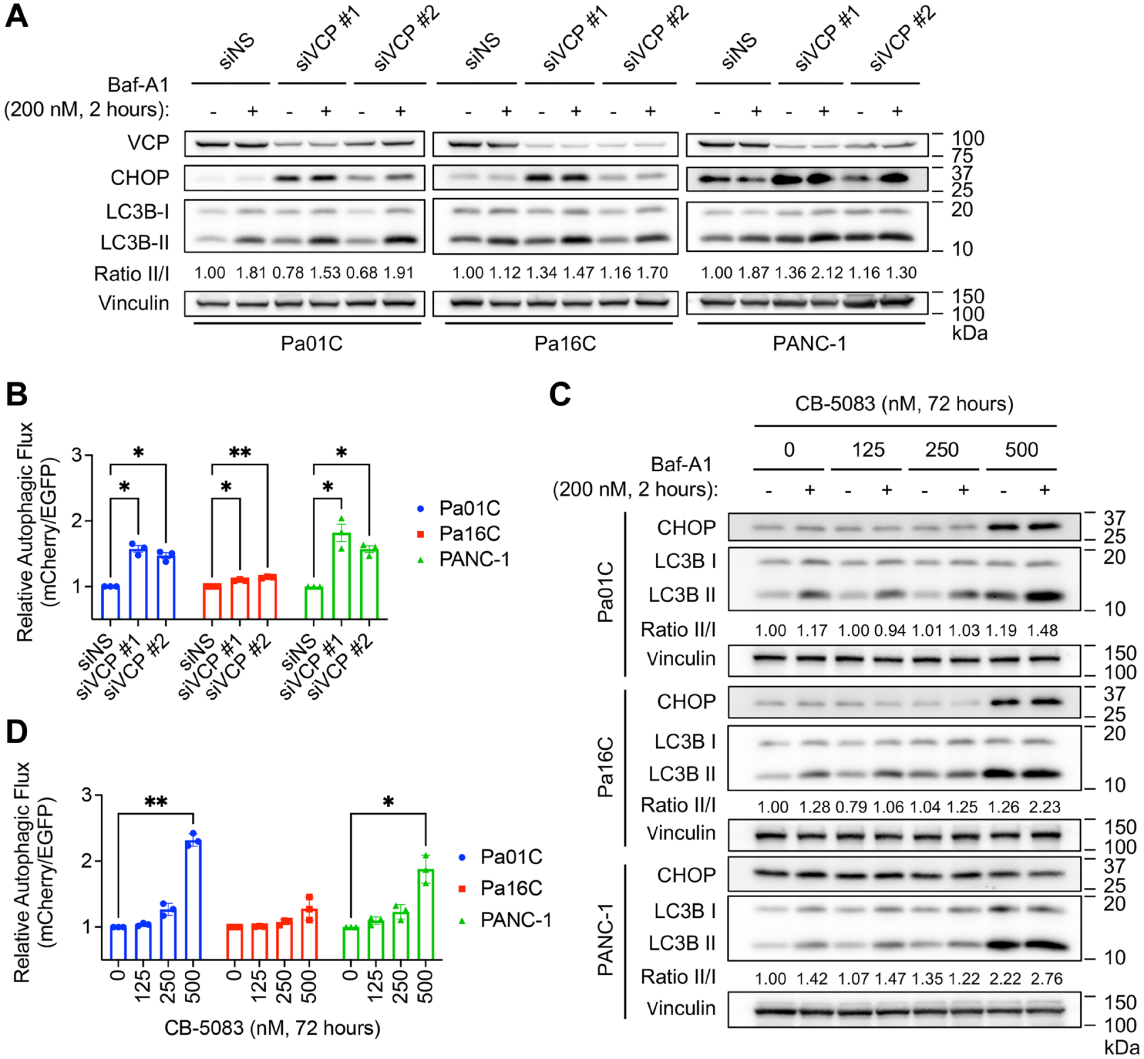
VCP loss elevates autophagic flux. (**A**) Cells were transfected for 72 hours with siNS, siVCP #1, or siVCP #2 and treated with bafilomycin A1 (Baf-A1, 200 nM) for 2 hours before lysate collection to inhibit autophagosome degradation. Immunoblot analyses were performed to determine the levels of indicated proteins. Autophagic flux was quantified by the ratio of LC3B-II to LC3B-I. Figure is representative of three biological replicates. (**B**) Cell lines stably expressing the mCherry-EGFP-LC3B reporter were transfected for 72 hours with siNS, siVCP #1, or siVCP #2. Autophagic index was determined using the ratio of mCherry to EGFP. Relative autophagic index was obtained by normalizing the autophagic index of treated groups to their respective controls. Data shown are the mean ± SEM of three independent experiments. Two-way ANOVA followed by Dunnett’s multiple comparison test was used to determine statistical significance between the different groups. ^*^p < 0.05 and ^**^p < 0.005. Knockdown efficiency is shown in Supplementary Figure 4B. (**C**) Cells were treated with VCP inhibitor CB-5083 for 72 hours at indicated concentrations and treated with bafilomycin A1 (Baf-A1, 200 nM) for 2 hours before lysate collection to inhibit autophagosome degradation. Immunoblot analyses were performed to determine the levels of indicated proteins. Autophagic flux was quantified using the ratio of LC3B-II to LC3B-1. Figure is representative of three biological replicates. (**D**) Cell lines stably expressing the mCherry-EGFP-LC3B reporter were treated with VCP inhibitor CB-5083 at the indicated concentrations for 72 hours. Autophagic flux was determined and analyzed for statistical significance as in panel B.

*KRAS*-mutant PDAC cells are highly addicted to autophagy [16, 17]. Accordingly, we and others have found that inhibiting autophagy may be a promising therapeutic approach for *KRAS*-mutant PDAC [16, 17]. VCP is an important contributor to autophagy in diverse contexts, although the precise mechanisms are unclear. To determine a potential role for VCP in regulating autophagy in *KRAS*-mutant PDAC, we utilized two methods to investigate changes in autophagic flux upon *VCP* suppression and inhibition. Both techniques monitor LC3B, a key component of the autophagy pathway [43]. First, we assessed autophagic flux via immunoblotting, using bafilomycin A1 (Baf-A1, 200 nM for 2 hours) to inhibit autolysosome acidification and autophagosome-lysosome fusion [44]. This method monitors the conversion of endogenous LC3B-I to the autophagosome-associated LC3B-II [16]. Interestingly, we observed that siRNA-mediated *VCP* suppression increased the ratio of LC3B-II to LC3B-I in Pa16C and PANC-1 cells, and this increase was either maintained or enhanced when autophagosome degradation was inhibited with bafilomycin A1 (Figure 5A).

To further assess autophagic flux, we utilized *KRAS*-mutant PDAC cell lines stably expressing the well-characterized autophagy flux reporter mCherry-GFP-LC3 [45]. Upon *VCP* suppression (Supplementary Figure 4A), there was a two-fold increase in autophagic flux in two of the three PDAC cell lines (Figure 5B, Supplementary Figure 4B). To complement genetic suppression of *VCP*, we also assessed autophagic flux in the presence of the pharmacological inhibitor of VCP, CB-5083. Akin to our findings with siVCP, the highest concentrations of CB-5083 increased the ratio of LC3B-II to LC3B-I (Figure 5C). We also observed a two-fold increase in autophagic flux upon VCP inhibitor treatment (CB-5083 at 500 nM for 72 hours) in two of three PDAC cell lines (Figure 5D, Supplementary Figure 4C), consistent with *VCP* suppression. These observations indicate that suppression or inhibition of VCP can upregulate autophagy in *KRAS*-mutant PDAC cell lines.

### Dual inhibition of VCP and autophagy enhances growth suppression and apoptosis

**Figure 6:**
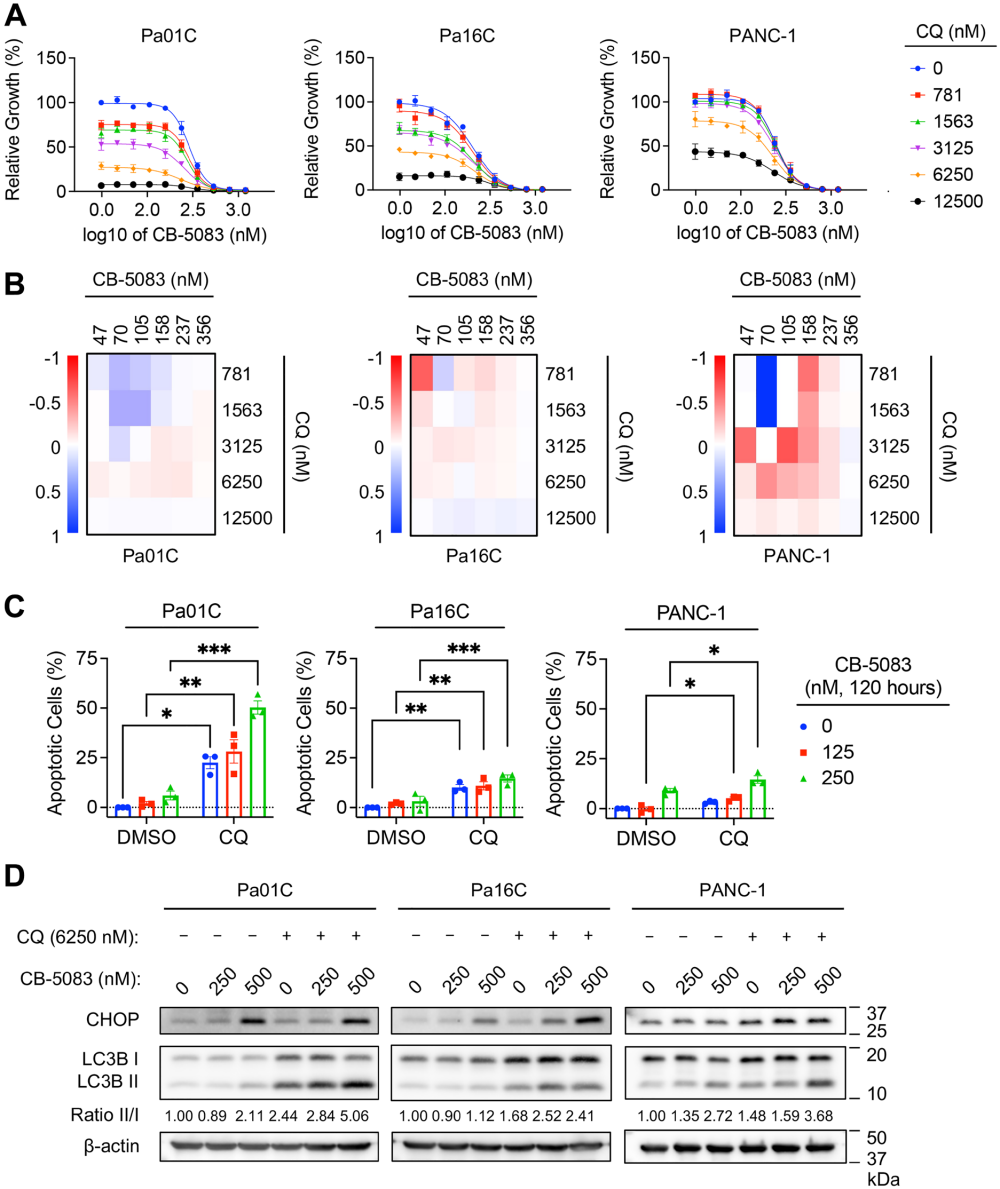
Dual inhibition of VCP and autophagy enhances growth suppression and apoptosis. (**A**) Cells were treated with a range of VCP inhibitor concentrations (CB-5083, 47–1,200 nM) and various constant concentrations of autophagy inhibitor CQ (781, 1563, 3125, 6250, 12500 nM). Proliferation was assessed by live cell counting. Data shown are the mean ± SEM of three independent experiments. (**B**) Heatmaps represent Bliss independence scores corresponding to representative growth curves shown in panel A. Bliss scores less than one (red) are synergistic, greater than one (blue) are antagonistic, and zero (white) indicates additivity. (**C**) Cells were treated with CB-5083 (125 or 250 nM) and CQ (6250 nM) for 120 hours. Percentage of cells undergoing apoptosis was determined by FACS analysis of Annexin-V and propidium iodide-labeled cells. All treated populations were normalized to their respective controls. Data shown are the mean ± SEM of three independent experiments. Two-way ANOVA followed by Šídák’s multiple comparisons test was used to determine statistical significance. ^*^p < 0.05, ^**^p < 0.005, and ^***^p < 0.001. (**D**) Cells were treated with VCP inhibitor CB-5083 (250 or 500 nM), autophagy inhibitor chloroquine (CQ, 6250 nM), or a combination of CB-5083 and CQ for 72 hours at the indicated concentrations. Immunoblot analyses were performed to determine levels of the indicated proteins. Autophagic flux was quantified using the ratio of LC3B-II to LC3B-I. Data shown are representative of three biological replicates.

We recently determined that inhibiting KRAS in *KRAS*-mutant PDAC stimulated a compensatory increase in autophagy that rendered them further addicted to autophagy and hypersensitive to autophagy inhibition [16]. We therefore hypothesized that the elevated levels of autophagy observed upon VCP inhibition may similarly result in a greater dependence on the autophagic process in VCPi-treated *KRAS*-mutant PDAC cells. If so, concurrent inhibition of VCP and autophagy should be more effective than either treatment alone. Although there are no specific inhibitors of autophagy in the clinic, chloroquine, which inhibits lysosomal acidification of the autophagosome and is thus an indirect inhibitor of autophagy, is routinely used [46]. We found that a range of VCP and autophagy inhibitor combinations enhanced chloroquine-mediated growth inhibition (Figure 6A). Bliss synergy analysis indicated that the combination had synergistic activity, indicated by values <0, in all three cell lines (Figure 6B). Additionally, the degree of apoptosis (Figure 6C, Supplementary Figure 5), and the induction of UPR as assessed by accumulation of CHOP (Figure 6D), caused by VCP inhibition were further enhanced by the combination treatment compared to VCPi alone. We conclude that concurrent inhibition of VCP and autophagy synergistically inhibits growth and induces apoptosis in *KRAS*-mutant PDAC cell lines.

## DISCUSSION

There is an unmet need for effective targeted therapies for *KRAS*-mutant PDAC. Attempts to address this need have included the pursuit of inhibitors of KRAS and its immediate downstream signaling pathways as well as of other vulnerabilities such as increased genomic instability and dependence on DNA damage repair pathways. For example, recent studies have indicated that targeting DNA damage response (DDR) proteins such as CHK1, WEE1, ATR, ATM, and PARP in PDAC may be efficacious [28–31]. However, an in-depth analysis of DDR proteins crucial for PDAC growth has not been carried out. To further explore DDR regulators, we performed a DDR-focused CRISPR-Cas9 loss-of-function screen and identified DDR proteins essential for *KRAS*-mutant PDAC growth. Our screen identified multiple targets of interest, including the pleiotropic AAA-ATPase VCP/p97, the protein arginine methyltransferase PRMT5, and the pleiotropic mitochondrial chaperone protein and transcriptional co-regulator PHB1/prohibitin 1. While the therapeutic potentials of inhibiting PRMT5 and PHB have been previously explored in PDAC [47, 48], such potential has been largely underexplored for VCP.

VCP is a multifunctional protein that has been shown to play critical roles in proteostasis (i.e., autophagy and UPS), cell cycle and DNA damage repair [34, 49–54]. Clinical studies have correlated VCP overexpression to advanced disease, metastasis, and worse patient outcome in many cancers [35, 55–57] including PDAC [37]. Thus, VCP has been identified as a potential therapeutic target for many cancer types. We found that both genetic suppression (siVCP) and pharmacological inhibition of VCP with CB-5083 (VCPi) limit cell viability, reduce clonogenic growth, and induce apoptosis in PDAC cell lines. Other preclinical studies have also shown that genetic suppression or pharmacologic inhibition of VCP limits cell viability and/or induces apoptosis in breast cancer [55], human choriocarcinoma [58], multiple myeloma [59], non-small cell lung cancer (NSCLC) [60], esophageal carcinoma [61], and ovarian cancer [62]. To further explore how VCP loss limits *KRAS*-mutant PDAC growth and induces apoptosis, we considered multiple mechanisms of VCP that may contribute to these effects.

One mechanistic basis for the growth suppressive and apoptotic effect of VCP loss likely involves the function of VCP in maintenance of proteostasis [63]. Upon treatment with siVCP or VCPi, we observed accumulation of ubiquitinated proteins in PDAC cell lines. Our result supports the importance of the well-established VCP function of maintaining proteostasis by extracting ubiquitinated proteins into the cytoplasm from the ER [34, 64, 65] and shuttling them to the proteasome for degradation [34, 66]. Similar to our findings, accumulation of ubiquitinated proteins has been observed in breast cancer upon VCP inhibition [55] and in multiple myeloma cell lines upon genetic suppression or inhibition of VCP [59]. Furthermore, we found that both siVCP and VCPi induced the unfolded protein response (UPR). This was expected, given that VCP loss leads to accumulation of ubiquitinated proteins in the ER, prompting activation of the UPR to maintain proteostasis [63]. In agreement, the aforementioned study exploring ERAD inhibition in PDAC found that a VCP-targeting tool compound, Eer1, induced UPR [67]. UPR activation has also been observed in various cancer types upon genetic suppression or pharmacologic inhibition of VCP [55, 59, 61, 62]. Given that unresolved ER stress and prolonged activation of UPR can result in apoptosis [63], our findings suggest that VCP supports PDAC growth in part by regulating protein degradation pathways to maintain proteostasis.

We had speculated that a second basis for growth suppression may be attributable to alterations in the cell cycle. Initially, VCP was identified as a cell cycle regulator in yeast, where VCP suppression yielded G2/M cell cycle arrest [68]. In human choriocarcinoma [58], NSCLC [60], and ovarian cancer [62], VCP inhibition resulted in G0/G1 cell cycle arrest. Contrary to these studies, VCP suppression had no effect on the cell cycle in human osteosarcoma [69]. Our studies in PDAC cell lines demonstrated heterogeneous outcomes with regards to cell cycle. We observed minor G0/G1 or G2/M cell cycle arrest in two PDAC cell lines (Pa01C and PANC-1) but no impact on cell cycle in a third (Pa16C). We conclude that cell cycle arrest is not a consistent mechanism by which VCP regulates the growth of KRAS-mutant PDAC cells.

A third mechanism whereby VCP may modulate PDAC growth is DNA damage repair. VCP has been shown to mediate DNA damage repair by facilitating DNA repair pathway decisions [70–72]. Consistent with a previous study showing that inactivation or depletion of VCP delays the resolution of DNA DSBs in bone and bladder cancer cells following radiation-induced DNA damage [52], we observed a modest accumulation of DSBs upon loss of VCP, indicating accumulation of unrepaired DNA damage. This demonstration that VCP mediates the resolution of DNA DSBs in *KRAS*-mutant PDAC cells supports another mechanism whereby VCP can be a potential therapeutic vulnerability in PDAC.

Finally, we sought to explore the regulation of autophagy by VCP in *KRAS*-mutant PDAC. The roles and mechanisms of VCP in autophagy are highly context dependent [73]. One recent study indicated that VCP is required for autophagy initiation in HeLa cells and another demonstrated its requirement for autophagosome maturation in muscle [51, 74]. In contrast, genetic loss of VCP increased autophagic flux in choriocarcinoma [58], indicating that VCP functions to negatively regulate autophagy in that context. In *KRAS*-mutant PDAC, we observed that both genetic and pharmacological loss of VCP can increase autophagic flux. This indicates that, like KRAS itself [16, 17, 75], VCP negatively regulates autophagy in *KRAS*-mutant PDAC, and is consistent with ER stress induction of autophagy as a pro-survival mechanism to restore proteostasis [76]. Similar to our recent finding that KRAS suppression caused a compensatory increase in autophagy [16], we propose that the increase in autophagic flux that we observed upon loss of VCP is a compensatory response to the suppression of ERAD pathways. In agreement, while our study was underway, another study explored endoplasmic-reticulum-associated [protein] degradation (ERAD) inhibition in PDAC using the ERAD inhibitor eeyarestatin I (Eer1), a VCP-targeting compound, and found that Eer1 limited PDAC cell viability [67].

Accordingly, we determined that combining VCPi with CQ enhanced anti-proliferative and pro-apoptotic effects in PDAC. This result parallels recent studies showing that concurrent inhibition of compensatory autophagy and the RAS-RAF-MEK-ERK signaling pathway was efficacious in PDAC growth inhibition [16, 17]. Moreover, the combination of VCPi and CQ enhanced the induction of UPR compared to either treatment alone. This suggests that ER-stress induced apoptosis observed upon inhibition of either VCP or autophagy alone can be further enhanced by inhibiting both regulators of ERAD.

In summary, our goal was to identify additional therapeutic targets for KRAS-driven PDAC. Our search was directed toward DDR proteins, stemming from our previous identification of this pathway as an important mechanism for PDAC survival [28]. We identified VCP as an important protein for PDAC growth and proteostasis via its regulation of protein degradation. VCP has therapeutic potential; however, explorations of this potential in preclinical studies were limited to the use of VCPi CB-5083. Although phase I clinical evaluation of CB-5083 was terminated due to an unexpected off-target ocular effect [77], a more selective analog (CB-5339) is currently under clinical evaluation in acute myeloid leukemia (NCT04402541) [78]. It will be interesting to evaluate CB-5339 or other clinical candidate VCP inhibitors in *in vivo* models of PDAC to support future clinical studies.

## MATERIALS AND METHODS

### Cell culture

Patient-derived xenograft human pancreatic cancer cell lines (Pa01C and Pa16C) were kindly provided by Dr. Anirban Maitra (MD Anderson Cancer Center). PANC-1 was obtained from the American Type Culture Collection (ATCC). PDAC cell lines (Pa01C, Pa16C, and PANC-1) stably expressing the mCherry-EGFP-LC3B reporter were generated as we have described previously [16]. We utilized cell lines that harbor the most prevalent mutations in PDAC, which include oncogenic *KRA*S G12D [79] and missense mutations of *TP53* [80]. These lines are also all wild type for *BRAF*, *PIK3CA* and *PTEN*. Despite having these driver mutations in common, it is well established that PDAC cancers and cell lines display considerable genetic heterogeneity [7]. For example, Pa01C and Pa16C are wild type for *CDKN2A* (p16), whereas PANC-1 has a *CDKN2A* deletion. Conversely, Pa16C and PANC-1 are wild type for *SMAD4*, whereas Pa01C has a *SMAD4* deletion. These and other genetic distinctions may influence how different biological activities respond to loss of VCP. All cell lines were cultured in Dulbecco’s Modified Eagle Medium (DMEM; Gibco) supplemented with 10% fetal bovine serum (FBS; Sigma-Aldrich), penicillin and streptomycin (Sigma-Aldrich) at 37°C in a humidified chamber with 5% CO_2_. All cell lines were validated via short-tandem repeat (STR) profiling and tested negative for mycoplasma contamination using the MycoAlert Mycoplasma Detection Kit (Lonza).

### Antibodies and reagents

The VCP inhibitor, CB-5083 (S8101), was from SelleckChem. The autophagy inhibitor, chloroquine diphosphate (CQ, 6628), the autophagosome-lysosome fusion inhibitor, and bafilomycin A1 (Baf-A1; Gibco, 19–148) were from Sigma-Aldrich. The following primary antibodies were used in this study: CHOP (2895), VCP (2648), LC3B (2775), and phospho-Histone H2A.X (Ser139, 9718) from Cell Signaling Technology. Lys48-specific ubiquitin antibody (05-1307) and vinculin (V9131) were from Sigma-Aldrich. The secondary antibodies, goat anti-rabbit IgG (31462) and goat anti-mouse IgG (31432), were from Invitrogen. pBABE-puro mCherry-EGFP-LC3B was a gift from Jayanta Debnath (Addgene plasmid #22418; http://n2t.net/addgene:22418; RRID: Addgene_22418) [45].

### DepMap analysis

Genetic dependency scores for KRAS, MYC, and VCP after CRISPR [81, 82] and shRNA [83] gene silencing were obtained from DepMap (21Q2 Public+Score, CERES). Mutation data for *KRAS* was also obtained from DepMap (21Q2). All pancreatic cancer-derived cell lines were included for analysis. More negative CERES scores indicate greater dependency on the indicated protein.

### Cloning of lentiCRISPRv2 vector

Blasticidin S deaminase (BSD) selection marker and a destabilization domain (DD) were cloned into lentiCRISPRv2 (Addgene, 52961) using NEBuilder HiFi DNA Assembly in a stepwise manner. Cloning of BSD was performed by assembling three components: two PCR-amplified pieces of lentiCRISPRv2 and one PCR-amplified piece of pLX304 (Addgene, 25890) with 13 bp overlaps. The lentiCRISPRv2 primers were used to remove the puromycin N-acetyltransferase selection marker. Cloning of the DD was performed by assembling two components: PCR-amplified piece of lentiCRISPRv2 BSD and PCR-amplified piece of DD-Cas9 with filler sequence and Venus (Addgene, 90085) with 25 bp overlaps. Small PCR products were purified using a PCR purification kit (QIAGEN, 28104) and large PCR products (> 6 kb) were run on a 1% agarose gel. Fragments were extracted using a gel extraction kit (QIAGEN, 28706) and were assembled by HiFi DNA Assembly according to manufacturer instructions (NEB, E2621).

### DNA damage response (DDR) CRISPR-Cas9 loss-of-function screen library design

The DDR CRISPR-Cas9 library consists of 6,040 sgRNAs targeting 504 genes that play a role in DNA repair, along with 1,000 non-targeting control sgRNAs. Each gene was targeted by ten domain-focused sgRNAs [84]. The CRISPR library oligonucleotides (74 nt) were synthesized by CustomArray, Inc. to have the same structure: a 5′ universal flanking sequence (GTGGAAAGGACGAAACACCG), a 20 nt target sgRNA, and a 3′ universal flanking sequence (GTTTTAGAGCTAGAAATAGCAAGTTAAAATAAGG). For details see Supplementary Table 1. The DDR library sgRNA pool was cloned into lentiCRISPR v2 using the NEBuilder HiFi DNA Assembly. PCR amplification of the sgRNA library pool was performed using the following primers:

**Table:** 

Array F:	TAACTTGAAAGTATTTCGATTTCTTGGCTTT ATATATCTTGTGGAAAGGACGAAACACCG
Array R:	ACTTTTTCAAGTTGATAACGGACTAGCCTT ATTTTAACTTGCTATTTCTAGCTCTAAAAC

The PCR reaction mixture consisted of the following: 2.5 μL of each primer (10 μM stock), 2 μL DDR library pool (0.2 μM stock), 18 μL molecular biology grade water, 25 μL 2X Q5 High-Fidelity 2X Master Mix (NEB, M0492L). The following PCR protocol was used: initial denaturation (98°C, 30 seconds), denaturation (98°C, 10 seconds), annealing (65°C, 30 seconds), extension (72°C, 25 seconds), final extension (72°C, 5 minutes). Denaturation, annealing, and extension steps were cycled 30 times. PCR products were purified using the MinElute PCR purification kit (QIAGEN, 28004). In parallel, the lentiCRISPRv2 (Addgene #52961) was digested with BsmBI (NEB, R0580) overnight at 55°C and then heat inactivated at 80°C for 20 minutes. Digestion products were run on a 0.8% agarose gel and a ~13 kb fragment was extracted using a gel extraction kit (QIAGEN, 28706).

Using 100 ng of BsmBI-digested lentiCRISPRv2 and 6.66 ng of the sgRNA library pool, a HiFi assembly reaction was performed. A HiFi assembly reaction mixture consisted of 100 ng BsmBI digested vector (2 μL), 6.66 ng DDR library pool (8 μL), and 10 μL HiFi DNA Assembly Master Mix (NEB, E2621). The reaction mixture was incubated for 1 hour at 50°C. For electroporation, 0.75 μL of HiFi assembly mixture was added to 25 μL of electrocompetent bacteria (Lucigen, 60242-2). The bacteria and DNA mixture was electroporated in ice-chilled cuvettes (Bio-Rad, 1652083) using Gene Pulser Xcell electroporator (Bio-Rad, 1652660) at 1800 Volts, 10 μFarad, 600 Ohm, and 1 mm cuvette gap. Recovery media (500 μL) of was added immediately post electroporation (Lucigen, 80026-1). Six electroporation reactions were performed to ensure high coverage of the entire library. Transformed bacteria was incubated for 1 hour at 37°C, plated on LB-ampicillin plates, and incubated overnight at 37°C. LB was added to each plate and transformed bacteria was removed using sterile scrapers. Cells from three plates were transferred to 500 mL LB cultures and incubated for 3 hours at 37°C. The cloned plasmid library was extracted using plasmid maxiprep kit (QIAGEN, 12362).

### DDR CRISPR-Cas9 screen

Lentivirus generation was performed as described previously [28, 39]. HEK293T cells were seeded in T75 flasks and allowed to attach overnight. Library plasmid (12 μg), psPAX2 (9 μg), pMD2.G (3 μg), and transfection reagent FuGENE6 (Roche) was suspended in Opti-MEM and incubated for 20 minutes at room temperature. The transfection mixture was added dropwise to the cells, centrifuged at 800 g for 1 hour, and incubated overnight. The following morning, harvest medium (DMEM with 20% FBS) was added and further incubated for 48 hours. Lentiviral supernatant was collected, concentrated using PEG-it™ virus precipitation solution (SBI), and cleared through a 0.45 μm filter. Viral titers were determined as previously described [85].

Human pancreatic cancer cell lines (5 × 10^5^ per well) were seeded in 6-well plates and incubated overnight. The next day, virus was added at an MOI of 0.2 [28]. The cells were selected with blasticidin (3 μg/mL) and Shield 1 ligand (1 μM) for 7 days. The infected cells were collected at the initial time point (Day 0), passaged every 3 days up to 28 days, and maintained at 1,000-fold coverage. The cells were collected at 14 and 28 days and flash-frozen in LN_2_. Three technical replicates were prepared for each cell line and time point. DNA was extracted using DNeasy Blood and Tissue Kit (QIAGEN). The DNA samples were run through two subsequent PCR reactions as we previously described [39]. Following the second reaction, the entire reaction was gel purified using a Gel Extraction Kit (QIAGEN) and purified and concentrated using ethanol precipitation. Finally, 3 pmol of DNA was loaded (PhiX spike of greater than 15%) onto an Illumina NextSeq 500 and sequenced with 75 bp, single-end reads. sgRNA abundances were quantified for each gene using MAGeCK analysis [86].

### siRNA transfections

Cells were reverse transfected using Lipofectamine™ RNAiMAX Transfection Reagent (Invitrogen, 13778150) and 10 nM of siRNA in Opti-MEM™ I Reduced Serum Medium (Gibco, 31985070). The non-specific siRNA (siNS, 4390844) and the first siRNA targeting VCP (siVCP #1, 4390824) were from Thermo Fisher. The second siRNA targeting VCP (siVCP #2) was from Horizon Discovery (J-008727-10-0005). Cells were seeded at varying densities depending on experimental procedure in 6-well plates in 2 mL of culture medium. Opti-MEM was equilibrated to room temperature and siRNAs were thawed on ice. Then, 2 μL of RNAiMAX was added to 98 μL of Opti-MEM. To suppress VCP, 110 nM of siRNA was added to Opti-MEM in a final volume of 100 μL. The siRNAs in Opti-MEM were added to the RNAiMAX/Opti-MEM mixture and incubated for 30 minutes at room temperature. The mixture (200 μL) was added dropwise to the well (2 mL) for a final concentration of 10 nM siRNA (2.2 mL total volume per well). Cells were incubated until each experimental time point was met. For all siRNA experiments, a non-specific control (siNS) was used, and knockdown efficiency of the target gene was confirmed by immunoblotting.

### Immunoblotting

For autophagic flux immunoblots, cells were treated with bafilomycin A1 (Baf-A1, 200 nM) 2 hours before lysate collection. Cells were washed with ice-cold PBS and lysed with RIPA lysis buffer (150 mM NaCl, 1% Triton X-100, 0.5% sodium deoxycholate, 0.1% SDS, and 50 mM Tris) supplemented with cOmplete™ protease inhibitor (Roche, 40091500) and phosphatase cocktail inhibitors (Sigma-Aldrich, 524625, 524624). Cells were incubated on ice for 10 minutes, scraped into microcentrifuge tubes, and centrifuged for 10 minutes at maximum speed (13,200 rpm) at 4°C. The supernatant was collected and protein concentration determined using the Pierce™ BCA Protein Assay Kit (Thermo Fisher Scientific, 23225) with bovine serum albumin (BSA; Sigma-Aldrich, A7906) as the standard.

Samples were prepared for SDS-PAGE with 4X Laemmli Sample Buffer (Bio-Rad; 1610747) and β-mercaptoethanol and boiled for 15 minutes before storage at −20°C. Equal amounts of protein per sample were loaded into polyacrylamide gels for separation of proteins and transferred onto PVDF membranes (Millipore, IPVH00010) activated in 100% methanol. Membranes were blocked in 5% non-fat milk diluted in TBST (TBS with 0.05% Tween-20) for 1 hour and washed with TBST. Membranes were incubated overnight in primary antibodies diluted in 5% BSA in TBST at 4°C. The membranes were then washed with TBST and incubated for 1 hour in secondary antibodies diluted in 5% non-fat milk in TBST. After washing with TBST, membranes were imaged using the ChemiDoc MP Imaging System and ECL western blotting substrates from Bio-Rad. Autophagic flux was measured using densitometric quantification of the LC3B doublet, and the ratio of LC3BII to LC3BI was reported.

### Anchorage-dependent proliferation

For siRNA experiments, 2.5 × 10^5^ cells were seeded in 6-well plates and transfected for 24 hours. Transfected cells were plated into 96-well (1 × 10^3^ cells per well) and 6-well plates (2.5 × 10^5^ cells per well) and allowed to attach overnight. Following overnight incubation, a Day 0 plate was quantified by counting calcein AM (500 nM, 30 minutes) labeled live cells using the SpectraMax i3x multimode detection platform (Molecular Devices) to assess plating efficiency. Following the Day 0 plate quantification, a 96-well plate was quantified every day for 5 days for growth tracking. To determine knockdown efficiency for the experiment, the 6-well plate was collected 72 hours after transfection and western blot analysis was performed. Raw cell numbers were adjusted accordingly by the plating efficiency as assessed on Day 0. Percentage viability was calculated by normalizing the adjusted treated cell counts to adjusted vehicle-control (siNS) samples. Technical replicates were averaged, and proliferation data depicted are representative of three biological replicates. Four-parameter drug response curves were generated using GraphPad Prism version 9.3.1.

For drug-treatment proliferation assays, cells were plated into 96-well plates (700–1,000 cells per well, depending on the cell line) and allowed to attach overnight. Following overnight incubation, a Day 0 plate was quantified by counting calcein AM (500 nM, 30 minutes) labeled live cells using the SpectraMax i3x multimode detection platform (Molecular Devices) to assess plating efficiency. For single drug studies, cells were treated with CB-5083 (47–1200 nM) and incubated for 72 or 120 hours before quantifying calcein AM labeled live cells. For synergy studies, cells were treated with CB-5083 (47–1200 nM) and chloroquine (781–12500 nM) in a dose-response matrix. After 120 hours of incubation, proliferation was quantified. For all drug-treatment proliferation studies, percentage viability was calculated as indicated previously. For all proliferation assays, BLISS synergy scores were analyzed as previously published [16].

### Anchorage-dependent colony formation

Cells were seeded at single-cell density (3.5–5 × 10^3^ cells per well, depending on the cell line) in 6-well plates. For siRNA genetic knockdown experiments, an additional knockdown confirmation plate was seeded (7–10 × 10^4^ cells per well, depending on the cell line) in 6-well plates and cells were reverse transfected on the same day. For pharmacological inhibition experiments, culture medium was replaced with medium containing CB-5083 (125–500 nM) or DMSO vehicle. For siRNA experiments, the knockdown efficiency plate was collected at 72 hours post reverse transfection and western blot analysis was performed. At 10–14 days after plating, the medium was carefully aspirated from all wells and cells were washed with PBS. Next, 1 mL of crystal violet in formaldehyde was added to each well and plates were incubated for 30 minutes at room temperature. Wells were washed gently with water and allowed to dry overnight at room temperature. Clonogenic colony formation was evaluated by imaging on a Typhoon™ FLA 7000 biomolecular imager. FIJI was used to analyze the percent cell coverage of the plate surface and all treated wells were standardized to their respective control wells (siNS or DMSO).

### Anchorage-independent growth in soft agar

Bacto-agar (1.8%), diluted 1:3 in phenol red-free DMEM+10% FBS, was used to form a base layer on either a 96-well plate (25 μL) or 24-well plate (250 μL). For the cell layer, 6% SeaPrep agarose (Lonza, 50302) was diluted to 2% with DMEM supplemented with 10% FBS and then combined 1:2 with cells resuspended in phenol red-free DMEM supplemented with 10% FBS to a final concentration of 1% SeaPrep agarose. Cells were plated (5,000 cells in 100 μL for 96-well plates or 50,000 cells in 1 mL for 24-well plates) and the agar was allowed to solidify for 10 minutes at 4°C. Even distribution of single cells at the time of plating was verified by microscopy. The following day, DMSO or VCPi was added to the top of each well. Colonies were allowed to grow for 14 days and evaluated periodically via microscopy. At day 14, 15 μL/well of alamarBlue (Thermo Fisher, DAL1025) was added to the 96-well plates and incubated for 4 hours prior to measurement of fluorescent signal (excitation 535 nM; emission 585 nM) on a SpectraMax i3. Each biological replicate included three technical replicates. Growth (%) was calculated by standardizing to DMSO wells, which were set to 100%, and growth curves were calculated as for anchorage-dependent proliferation. Three biological replicates were averaged for each treatment condition. Also on day 14, images were collected from the 24-well plates for all three biological replicates, using a 10X objective on an EVOS 5000 microscopy. Representative images were selected for figure panels.

### Flow cytometry assays

For apoptosis assays, R&D Systems™ TACS Annexin V-FITC Apoptosis Detection Kit (4830-250-K) was used to measure apoptosis according to the manufacturer’s instructions. Detached cells in the spent culture medium and trypsinized cells were collected and centrifuged at 300 g for 5 minutes. Cells were washed with PBS and centrifuged at 300 g for 5 minutes before incubating in the Annexin V Incubation Reagent (1% Annexin V-FITC and 1x propidium iodide solution in 1x calcium-containing binding buffer) in the dark for 15 minutes. The cell mixture was diluted 1:5 in 1x calcium-containing binding buffer and analyzed using a BD LSRFortessa flow cytometer. Over 2 × 10^4^ cells were collected and exported using FACSDiva v8.0.1. Using Cytobank, a FSC-A (x) versus SSC-A (y) plot was used to exclude debris and generate a “cells” gate for intact cells. “Cells” were plotted in a FITC-A (x) versus PI-A (y) plot and apoptotic cells (FITC+) were analyzed. Percentage of apoptotic cells was normalized by subtracting the percentage of apoptotic cells from the vehicle control (siNS or DMSO) from treated samples.

For cell cycle analysis, adherent cells were washed with PBS, trypsinized, and centrifuged at 300 g for 5 minutes. The cell pellets were fixed with 1% paraformaldehyde in PBS, incubated for 15 minutes on ice, centrifuged as previously indicated, resuspended gently in 70% ethanol and incubated at 4°C for at least 2 hours. Fixed cells were then pelleted, washed with PBS, resuspended in 40 μg/ml propidium iodide (PI) and 100 μg/ml RNase A in PBS, and incubated at 37°C for at least 1 hour. Cells were analyzed using a BD LSRFortessa flow cytometer and over 20,000 cells were exported using FACSDiva v8.0.1. Cell cycle analysis was then performed using FCS Express. A “cells” gate was established using a FSC-A (x) versus SSC-A (y) plot and a “singlets” gate was established using a FSC-A (x) versus FSC-H (y) plot. Singlets were then analyzed via a histogram for PI-A content prior to employing the Multicycle algorithm to analyze cell cycle.

To quantify autophagic flux, adherent cell lines stably expressing the mCherry-EGFP-LC3B reporter were washed with PBS, trypsinized, and suspended in growing medium. The cells were analyzed using a BD LSRFortessa flow cytometer. Over 20,000 cells were analyzed and autophagic index was quantified using the ratio of mCherry to EGFP. Statistical analyses were performed using GraphPad Prism version 9.3.1. The statistical significance between experimental groups were determined using the two-way ANOVA followed by Dunnett’s multiple comparison test.

### Immunofluorescence, imaging, and analysis

Cells were plated on 12-well glass-bottom dishes (MatTek Corporation, P12G-1.5-14-F) and treated for 24 hours as specified in the Figure or Figure legend. Treated cells were washed with PBS and fixed in 4% paraformaldehyde in PBS for 20 minutes, then washed in PBS and either stored in 0.04% sodium azide in PBS at 4°C or permeabilized with 0.5% Triton X-100 in PBS for 5 minutes, washed with PBS, blocked in 10% normal goat serum (NGS) and 2% BSA in PBS for 30 minutes, and incubated in antibodies targeting γH2AX (1:100 in 5% NGS and 1% BSA in PBS) for 2 hours. Cells were washed in PBS, incubated in goat anti-rabbit Alexa Fluor 488 secondary antibody (1:100 in 1% BSA in PBS) for 45 minutes, counterstained with DAPI (1:10,000 in PBS) for 10 minutes, and washed with PBS. Fixed cells were imaged on an EVOS M7000 wide-field microscope with a 40X, 0.65 NA objective.

Background was removed from each image using a sliding paraboloid on FIJI [87]. Background-subtracted images were then analyzed using Cell Profiler [88]. The primary objects (nuclei) were identified using the DAPI images and the integrated density of γH2AX was determined for each nucleus using the Alexa-488 images. Nuclei touching the edge were discarded from analyses. For each biological preparation, the mean value of γH2AX integrated density of the DMSO control was calculated. All nuclei from each condition were divided by that value. No difference was found between the two biological preparations for the relative integrated density of γH2AX; therefore, all nuclei were grouped together for statistical analysis. Statistical analyses were performed using GraphPad Prism version 9.3.1 and outliers were removed using *Q* = 0.1, ROUT [89]. Significance was evaluated using a one-way ANOVA and Kruskal-Wallis test relative to DMSO.

### Statistical analysis

All statistical analyses were performed using built-in tests within GraphPad Prism version 9.3.1. The statistical method utilized for each Figure is noted in the corresponding Figure legend. Error bars indicate mean ± SEM for *n* ≥ 3 independent experiments unless noted otherwise. *P*-values are denoted within each Figure legend.

## SUPPLEMENTARY MATERIALS





## Abbreviations

ANOVA: analysis of variance
ATCC: American Type Culture Collection
ATM: ataxia telangiectasia mutated
ATR: ataxia telangiectasia and rad3-related protein
BCA: bicinchoninic acid
BSA: bovine serum albumin
BSD: blasticidin S deaminase
CHOP: C/EBP homologous protein
CQ: chloroquine
CRISPR: clustered regularly spaced interspaced short palindromic repeats
DD: destabilization domain
DDR: DNA damage response
DMEM: Dulbecco’s modified Eagle’s medium
DMSO: dimethyl sulfoxide
ER: endoplasmic reticulum
ERAD: endoplasmic-reticulum-associated protein degradation
FBS: fetal bovine serum
GFP: green fluorescent protein
HR: homologous recombination
IF: immunofluorescence
IgG: immunoglobulin G
MOI: multiplicity of infection
NGS: normal goat serum
NCI: National Cancer Institute
NHEJ: non-homologous end joining
NIH: National Institutes of Health
NSCLC: non-small cell lung cancer
PARP: poly adenosine diphosphate-ribose polymerase
PDAC: pancreatic ductal adenocarcinoma
PHB: prohibitin
PRMT5: protein arginine methyltransferase 5
PVDF: polyvinylidene fluoride
RIPA: radioimmunoprecipitation assay
STR: short-tandem repeat
TBST: TBS with 0.05% Tween-20
UPR: unfolded protein response
UPS: ubiquitin-proteasome system
VCP: valosin-containing protein
VCPi: valosin-containing protein inhibitor
