# Supplementary material for: VCP/p97, a pleiotropic protein regulator of the DNA damage response and proteostasis, is a potential therapeutic target in KRAS-mutant pancreatic cancer
Source: Genes Cancer. 2023 Mar 10;14:30–49. doi: 10.18632/genesandcancer.231 (PMC10010283; doi:10.18632/genesandcancer.231)
Supplement: Supplementary file 1 [file ganc-14-231-s001.pdf]

VCP/p97, a pleiotropic protein regulator of the DNA damage response and proteostasis, is a potential therapeutic target in *KRAS*-mutant pancreatic cancer

SUPPLEMENTARY MATERIALS

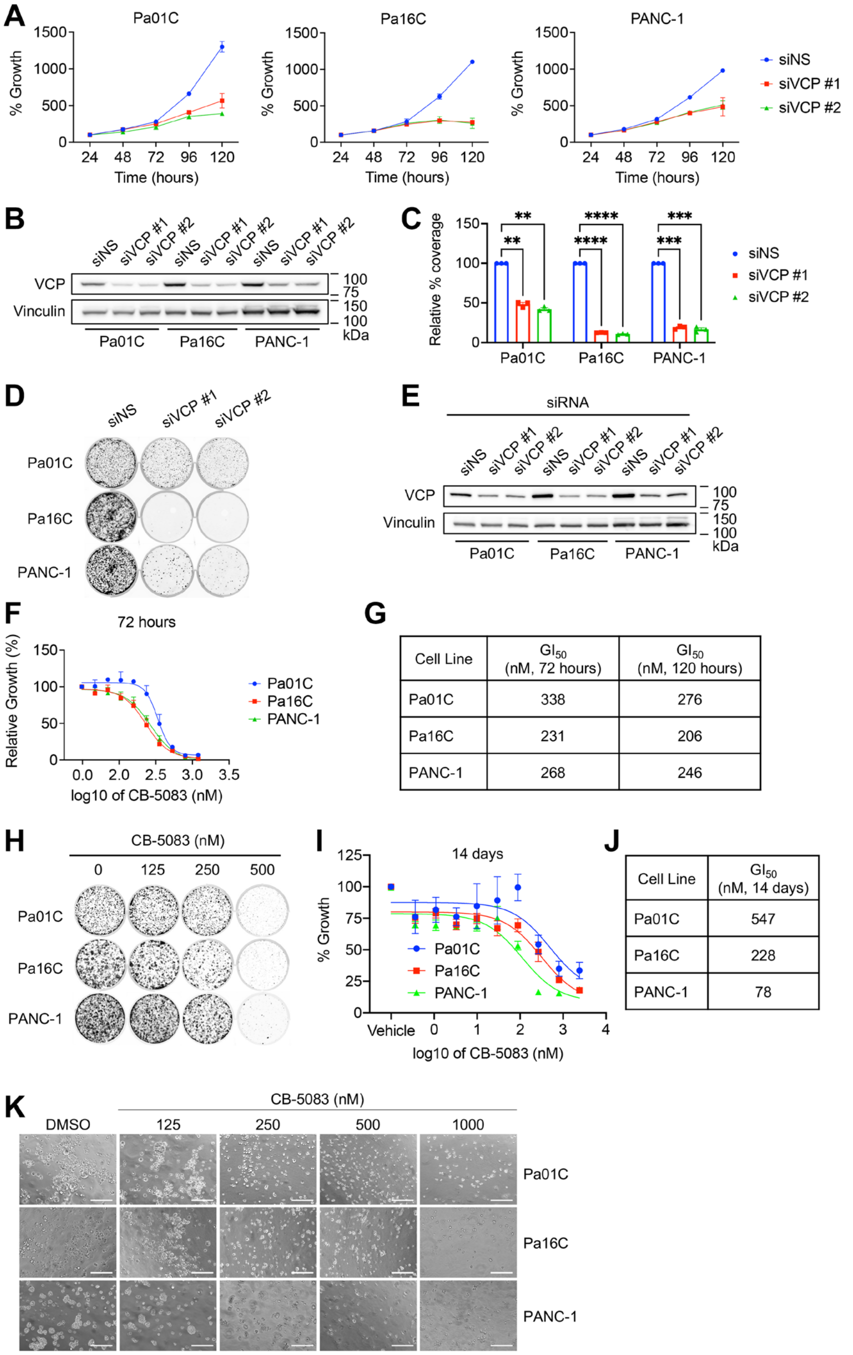

Supplementary Figure 1: VCP is required for PDAC growth and polyubiquitinated protein degradation. (**A**) Cells were transiently transfected with nonspecific (siNS) or *VCP*-targeting siRNAs (siVCP #1, siVCP #2) for the indicated times. Proliferation was assessed by live cell counting at 24-hour intervals for 120 hours. Mean cell counts were normalized to 24 hours post-transfection. Data shown are the mean ± SEM of three independent experiments. Knockdown efficiency is shown in panel B. (**B**) Cells from panel

A were immunoblotted for VCP protein. Vinculin served as a loading control. Figure is representative of three biological replicates that correspond to Figure 2B and panel A. (**C**) Anchorage-dependent colony-forming capacity was evaluated by staining with crystal violet 10–14 days after reverse transfection with siNS or *VCP*-targeting siRNAs. (**D**) Representative images of three biological replicates of assays quantitated in panel C. (**E**) Immunoblot analyses of knockdowns from cells in panels C and D. Figure is representative of three biological replicates that correspond to Figure 2C and to panel C. (**F**) Cells were treated with a range of VCP inhibitor (CB-5083) concentrations (47–1200 nM) and proliferation was assessed by live cell counting at 72 hours. Data shown are the mean ± SEM of three independent experiments. (**G**) Mean GI_{50} from cells treated for 72 hours (shown in panel F) and 120 hours (curves not shown). (**H**) Anchorage-dependent colony-forming capacity was evaluated by staining with crystal violet 10–14 days after treatment with CB-5083 (125, 250, and 500 nM). (**I**) Anchorage-independent proliferation was evaluated by alamarBlue staining of colonies formed after 14 days of growth in soft agar in the presence of a range of concentrations of CB-5083 or DMSO vehicle. Data shown are the mean ± SEM of three biological replicates, each of which included three technical replicates. (**J**) GI_{50} for each cell line depicted in panel I. (**K**) Representative images for additional inhibitor concentrations, representative of three biological replicates and three technical replicates each, corresponding to Figure 2E. Scale bar, 300 μm.

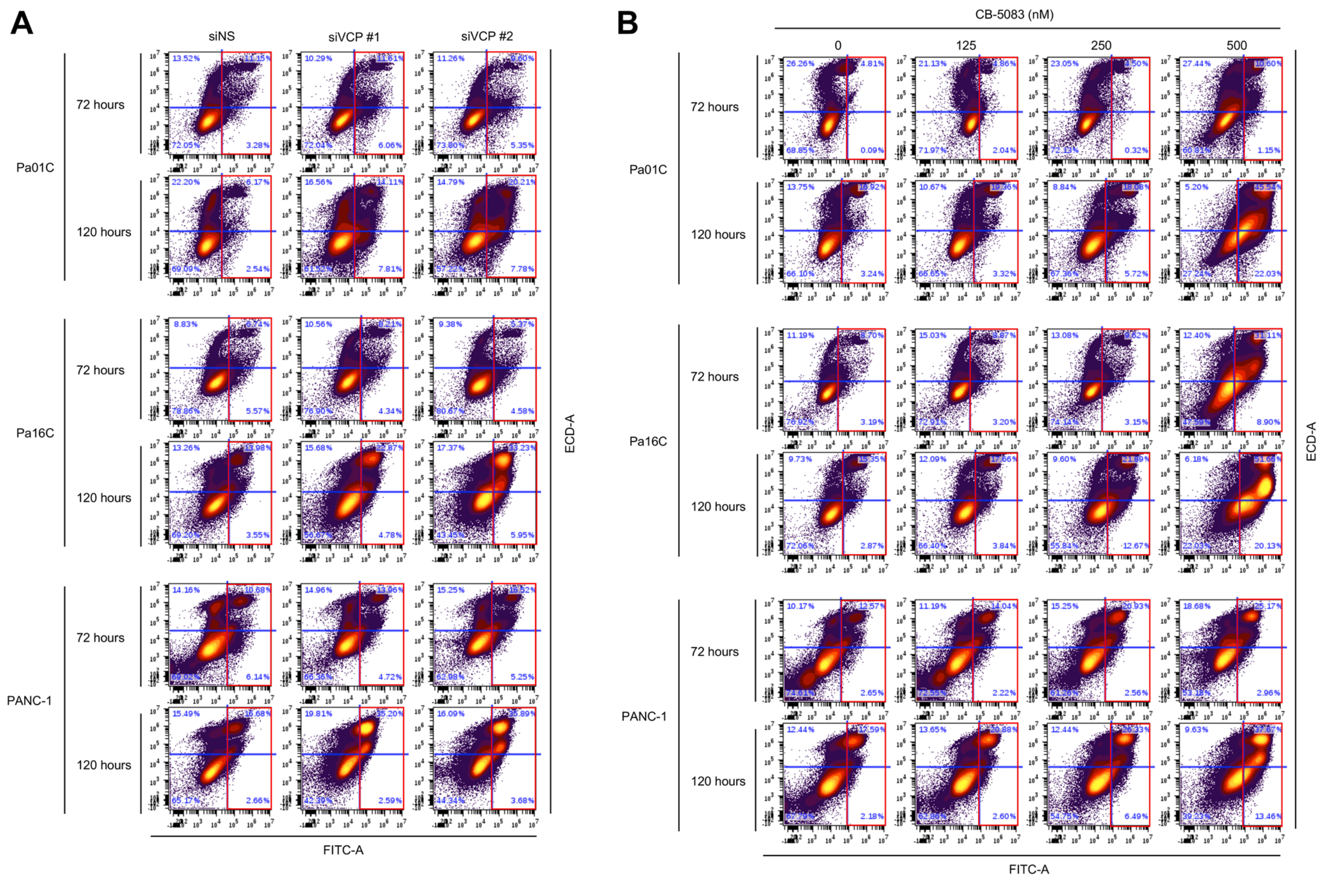

Supplementary Figure 2: Loss of VCP induces PDAC cell death. (**A**) Cells were reverse transfected with non-specific control or *VCP*-targeting siRNAs for 72 or 120 hours. Percentage of cells undergoing apoptosis was determined by FACS analysis of Annexin-V and propidium iodide-labeled cells. Representative flow cytometry plots depict gating and percent apoptosis for each treatment. Knockdown efficiency is shown in Figure 3A. (**B**) Cells were treated with CB-5083 (125, 250, or 500 nM) for 72 or 120 hours, and analyzed as in panel A.

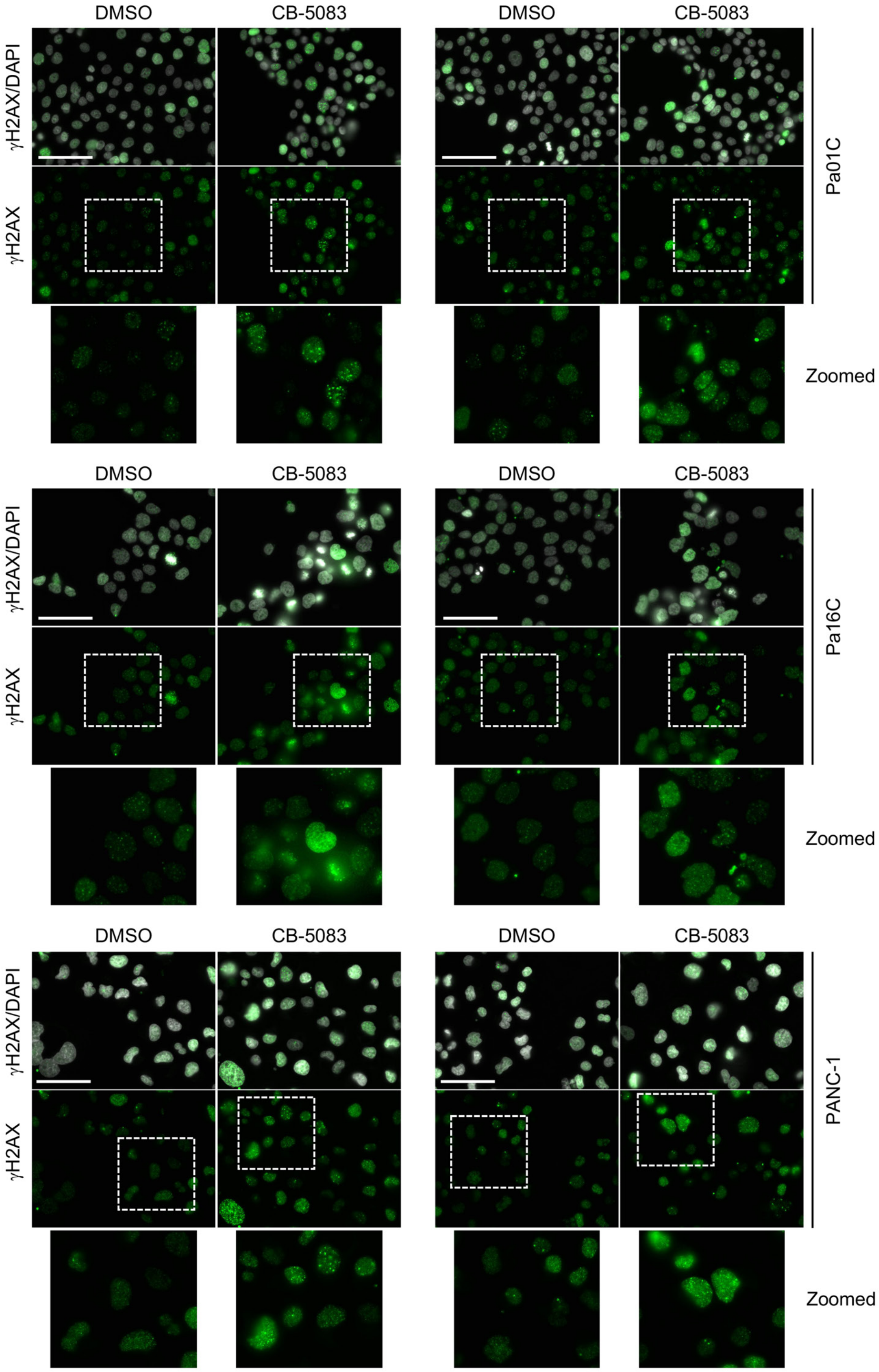

Supplementary Figure 3: VCP, a regulator of DDR, helps mediate DNA damage repair in PDAC. Additional representative images of immunofluorescence (IF) to monitor γH2AX expression (green) and nuclei (white) in PDAC cell lines upon treatment with DMSO or CB-5083 (500 nM, 24 hours). Scale bar, 75 μm.

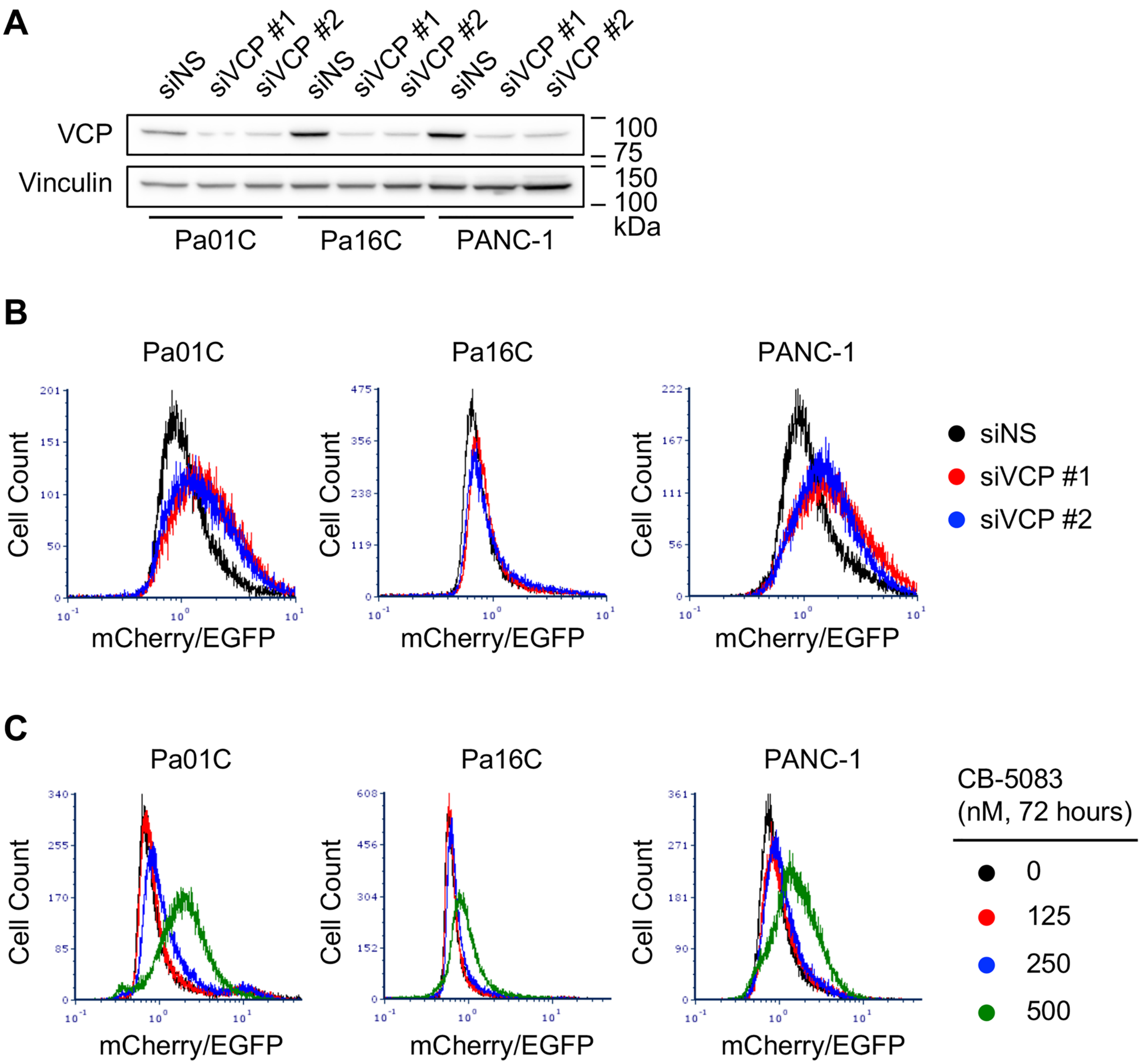

Supplementary Figure 4: VCP loss elevates autophagic flux. (**A**) Cell lines stably expressing the mCherry-EGFP-LC3B reporter were transiently transfected with siNS, siVCP #1, or siVCP #2 for 72 hours. Immunoblot analyses were performed to determine knockdown efficiency. Figure is representative of three biological replicates that correspond to Figure 5B and panel B. (**B**) Flow cytometry plots for cell lines from panel A depict cell count and the mCherry/EGFP ratio for each treatment. Plots are representative of three independent experiments. (**C**) Cell lines stably expressing the mCherry-EGFP-LC3B reporter were treated with VCP inhibitor CB-5083 at the indicated concentrations for 72 hours. Flow cytometry plots depict cell count and the mCherry/EGFP ratio for each treatment. Plots are representative of three independent experiments.

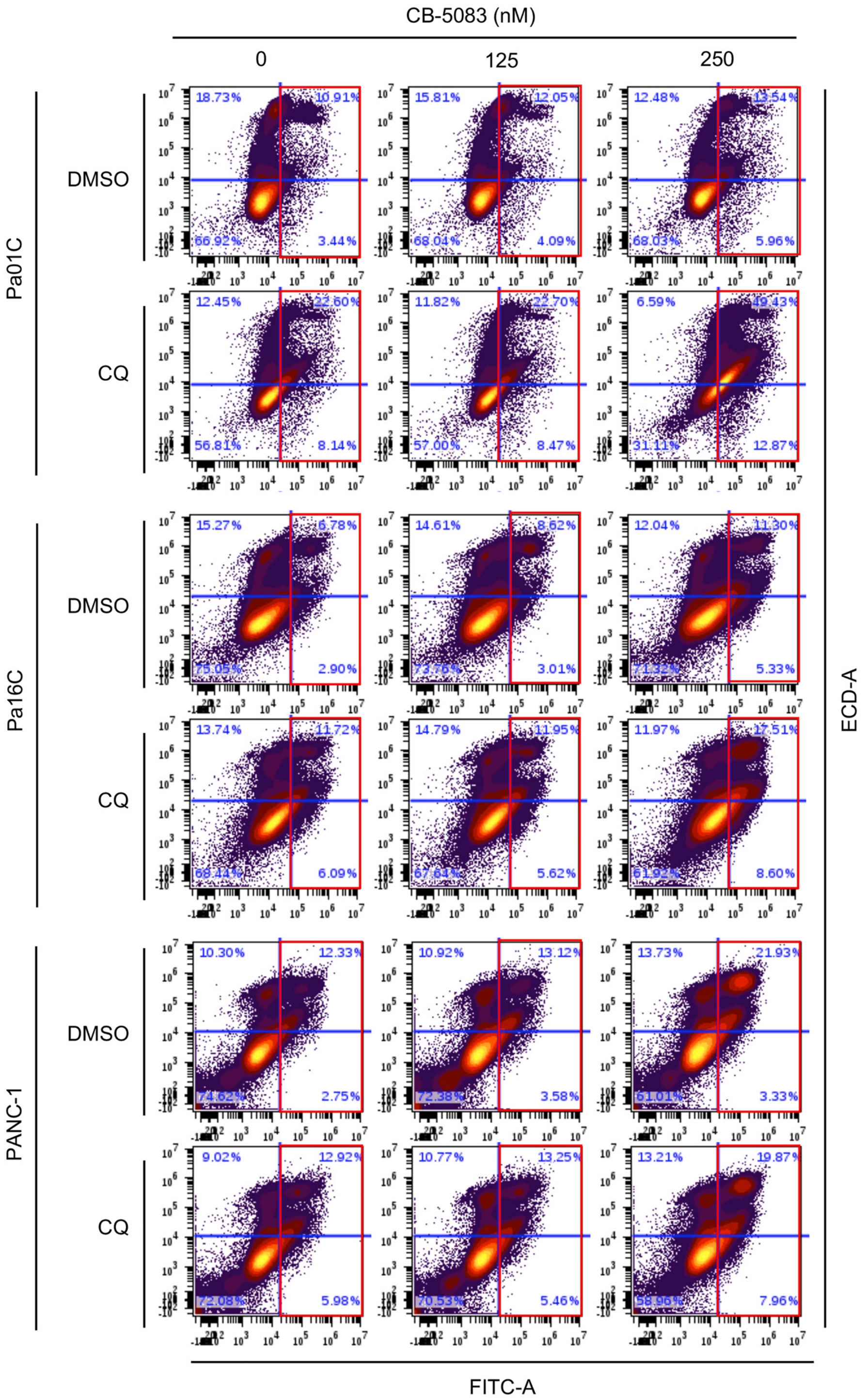

Supplementary Figure 5: Dual inhibition of VCP and autophagy enhances growth suppression and apoptosis. PDAC cell lines were treated with CB-5083 (125 or 250 nM) and CQ (6250 nM) for 120 hours. Apoptosis was determined by FACS analysis of Annexin-V and propidium iodide-labeled cells. Representative flow cytometry plots depict gating and percent apoptosis for each treatment.

Supplementary Table 1: Detailed DDR CRISPR-Cas9 guide sequences. See Supplementary Table 1
